# The influence of changing fire regimes on specialized plant–animal interactions

**DOI:** 10.1098/rstb.2023.0448

**Published:** 2025-04-17

**Authors:** Felicity E. Charles, April E. Reside, Annabel L. Smith

**Affiliations:** ^1^ School of the Environment, Faculty of Science, The University of Queensland, Saint Lucia 4072, Queensland, Australia

**Keywords:** ecological specialization, evolutionary ecology, feedback, fire ecology, global change, niche breadth

## Abstract

Ecological effects of changing fire regimes are well documented for plant and animal populations, but less is known about how fire influences, and is influenced by, specialized plant–animal interactions. In this review, we identified mutualistic (pollination, seed dispersal and food provision), commensal (habitat provision) and antagonistic (seed predation, herbivory and parasitism) plant–animal interactions from fire-prone ecosystems. We focused on specialized interactions where a single genus depended on one to two genera in a single family of plant or animal. We categorized the plant partner’s post-fire reproductive mode to assess the likely outcome of changing fire regimes on ecological functions provided by these interactions. Traits underlying specialization in fire-prone ecosystems for plants were: post-fire reproductive mode, time to maturity, morphology and phenology; and, for animals: dispersal, specialized organs, nesting and egg deposition substrates, plant consumption behaviours and pollinator behaviours. Finally, we identified a number of cases where stabilizing feedbacks maintained plant–animal interactions under natural fire regimes. Potential reinforcing feedbacks were also identified, but were more likely to happen abruptly and result in collapse of the plant–animal partnership, or partner switching. Our synthesis reveals how fire regime changes impact fire-dependent specialist plant–animal interactions and potentially drive eco-evolutionary dynamics in fire-prone ecosystems globally.

This article is part of the theme issue ‘Novel fire regimes under climate changes and human influences: impacts, ecosystem responses and feedbacks’.

## 1. Introduction

Fire plays an important role in the evolution of plant and animal traits and in the functioning of ecosystems [1–3], but contemporary changes in fire regimes are driving global biodiversity declines [4–6]. Rapid changes in land use and climate are increasing the frequency, intensity and duration of fires in many parts of the world, especially at mid to high latitudes [7–9]. In other regions, a reduction in cultural or prescribed burning, coupled with high-biomass invasive species [10,11], has changed spatial patterns of wildfire risk [12,13]. These changes have led to increasing large, catastrophic wildfires in many regions globally [14,15]. How species and ecosystems will respond to these rapid changes is not well known, especially for specialists that have specific resource requirements (e.g. a single genus of plant or animal relying on one to two genera within a single family for pollination, dispersal, food or habitat [16,17]) [18,19].

In contrast to rapid contemporary changes, the ecological and evolutionary effects of historical fire regimes have been well documented for a range of plant and animal populations and ecological communities [5,20–22]. Spatio-temporal patterns of biodiversity in fire-prone ecosystems are shaped by the interplay between plant and animal functional traits, life history parameters, environmental variation and fire regimes (characterized by frequency, severity, size and season) [21–24]. In fire-prone ecosystems, plant population dynamics are influenced by morphological traits such as flammability [25,26], branch retention or shedding [25], and bark thickness [25]; by reproductive traits including post-fire reproductive mode, post-fire flowering and serotiny [22,27,28]; and by life history parameters including survival [27] and recruitment [29–31] rates. For animals, key factors influencing fire-related population dynamics include survival, habitat requirements, movement, dispersal and behaviour (e.g. taking refuge in burrows or rock fissures) [23,27,28]. A vast body of literature has documented how fire-induced changes in plant populations affect animals, such as structural changes in post-fire vegetation driving succession in animal abundance [32–35]. Similarly well known are the directional effects of animals on vegetation structure and how these influence, and are influenced by, fire (e.g. grazing animals modulating the fire regime through their influence on vegetation structure [34,36]). Less is known, however, about how variation in fire regimes affects specialized plant–animal interactions, where one genus is dependent on one to two genera in a single plant or animal family for pollination, dispersal, food or habitat (e.g. [37]).

Understanding the effects of fire regimes on specialist interactions is important for at least two reasons. First, theory predicts that, while generalists have wider environmental tolerances [38] and often complex genetic structure [39–41], specialists may evolve faster than generalists because they have a simpler ‘fitness landscape’ for a given environmental niche [42–44]. Specialists can more rapidly fix alleles that increase their fitness for a given niche, while generalists require longer time scales [42]. Environmental variability can, therefore, pose a stronger selection pressure on specialists than generalists owing to their restricted niche [45,46]. In the context of rapidly changing fire regimes, eco-evolutionary feedbacks might, therefore, be stronger for specialist than generalist interactions [42,47,48].

The second reason why understanding fire effects on specialist plant–animal interactions is important is that specialists have a higher risk of extinction under rapid environmental change than generalists [19,49,50]. Although specialists have potential for rapid adaptation, their ability to do so depends on how their adaptation potential co-varies with the direction, scale and rate of environmental change [42,46,47]. Furthermore, the interaction type (i.e. mutualism, commensalism or antagonism) could influence the evolutionary outcome and, thus, the risk of extinction [51,52]. For example, co-dependencies between interactors in mutualisms might make both interactors more susceptible to extinctions [53]. ‘Partner switching’, whereby one interaction partner switches to interact with a new interaction partner [51], and changes in interaction type, such as commensalisms or mutualisms evolving into antagonisms [51,52], have been documented under rapid environmental change. Whether partner or interaction type switching has happened for specialized interactions in the context of changing fire regimes is largely unknown. Thus, gaining greater understanding of how changing fire regimes will impact specialist interactions and how these changes might feed back into ecological changes will improve our ability to manage ecosystems.

In this study, we examined the influence of fire regimes on specialized plant–animal interactions. We first compiled a database of specialized plant–animal interactions from fire-prone ecosystems to identify cases where changing fire regimes might drive eco-evolutionary dynamics. In some cases, a species was identified as being generalist across its distributional range but specialized in a particular ecosystem where its main resource was abundant (e.g. 50% of its diet comprised a single family of plants [54]) [55,56]. We included such cases in our review. We characterized plant–animal interactions as mutualistic, commensal or antagonistic [52,57] and then applied a framework for classifying plant post-fire reproductive mode [22]. This allowed us to synthesize a wide range of ecological processes driven by specialized plant–animal interactions and to explore potential outcomes of changing fire regimes for these processes. We then reviewed traits involved in these specialist plant–animal interactions to identify evidence of natural selection or evolution on ecological time scales. This synthesis allowed us to draw some conclusions about how changes in fire regimes might influence eco-evolutionary feedback in specialist plant–animal interactions. This information will help us understand the dynamics of tightly coupled relationships that are affected by fire and will inform planning of appropriate management and conservation interventions.

## 2. Plant–animal interactions in fire-prone ecosystems

We searched the literature to identify specialist interactions (i.e. a single genera dependent on one to two genera in a single family of plant or animal for pollination, dispersal, food or habitat) which were mediated by fire. Given the specificity and infancy of this research topic, our review was semi-systematic, combining formal literature searches with information found through our general reading. Using Web of Science on 15 October 2023 and Scopus on 26 October 2023, we searched the literature using the following terms: ecolog*, enviro*, fire, specialist interaction, plant–animal interaction, plant, animal, commensal, mutual* interaction and antagonist* interaction, pollinat*, herbiv*, predat*, eco-evolution* and eco-evolutionary dynamics. Results from Web of Science revealed that specialist insect interactions beyond pollination were not being captured in these search terms. We thus conducted a second search on both platforms for consistency across the databases on 26 October 2023 using the search strings: ‘fire AND specialis* AND insect’ and ‘fire AND specialist AND larva*’.

Our searches retrieved 358 articles from Web of Science and 212 articles from Scopus, with no overlap between the databases. Titles and abstracts were screened using revtools [58] in R 4.3.1 [59] to filter only empirical data articles that explicitly analysed the effects of fire and specifically mentioned a plant–animal interaction. A study might have examined effects of grazing and fire grouped as ‘disturbance’ but was only included in our review if fire effects could be separated from other disturbances. Title screening resulted in 157 articles from Web of Science and 47 articles from Scopus. Abstracts were then screened for the same criteria, resulting in 62 articles from Web of Science and 16 from Scopus. Whole-article screening was then performed to ensure that the plant–animal interactions fitted our definition of a specialized interaction and the species involved in the interaction were named in the article, giving a final set of 25 articles.

**Figure 1.**
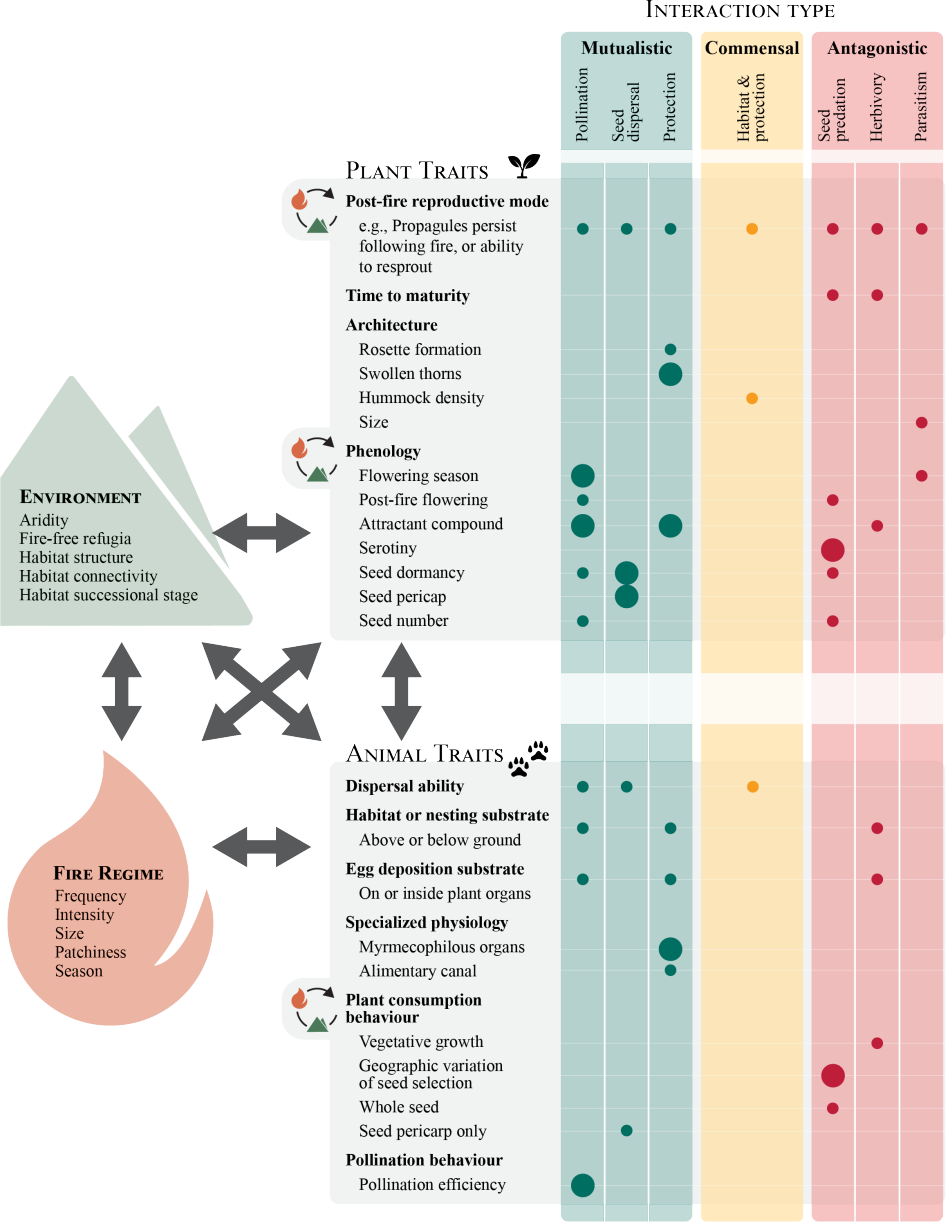
Plant and animal morphological, behavioural and reproductive traits involved in specialized plant–animal interactions in fire-prone ecosystems. Points indicate traits identified from the literature as being involved in different types of interactions. The relative potential for selection to act on traits and feed back into ecological dynamics is signified by the size of the points: small points indicate traits likely under weak selection and large points indicate traits potentially under strong selection. Environmental variation influences these interactions and the fire regime by controlling habitat structure, connectivity and aridity. The fire regime controls the environment by consuming vegetation and influencing recruitment and mortality processes in plants and animals. Plant and animal traits, particularly plant post-fire reproductive mode and morphology, and plant consumption behaviours in animals, are likely to feed back into the environment and fire regime. For example, herbivory affects habitat connectedness, which in turn affects the size, patchiness and intensity of fires, feeding back into population dynamics of herbivores. Pollinators affect habitat structure by influencing flowering season, rate of seed set and, therefore, the potential for plant recruitment. This feeds back into the fire regime by influencing biomass and post-fire succession, with subsequent influences on fire frequency.

Our review was complicated by the fact that plant–animal interactions or specialist interactions are rarely framed as such in fire ecology literature. For example, the Australian mallee emu-wren is dependent on grasses in the genus *Triodia* (Poaceae) and has been nearly driven to extinction by changing fire regimes and habitat loss [60]. This is rarely described as a specialist plant–animal interaction because *Triodia* is so widespread and commonly described only in terms of being habitat for the bird [61]. Thus, we added to the list from the systematic search 24 articles of which we were aware through our general reading (electronic supplementary material [62], table S1). Specialist plant–animal interactions were assessed on a case-basis, drawing upon multiple articles where available, rather than on a publication basis (i.e. referring to only the most recent study of the interaction). In order to identify how changing fire regimes might impact these interactions, we classified key plant and animal traits that contributed to their persistence and the strength of selection on these traits (figure 1).

Our aim was to understand how changing fire regimes would impact plant–animal interactions, but the fire ecology of species in each study was not always described and, in many cases, might be unknown. Plants are the foundation of animal habitat, and understanding plant responses to fire was necessary to identify potential changes to plant–animal interactions. Therefore, we conducted additional research on each case study to classify the plant species in the interaction by its post-fire reproductive mode [22]—one of the most important traits determining plant fire responses. For most cases, we retrieved this information from the TRY plant database [63], AusTraits [64], BROT [65] or the Fire Effects Information System [66] (electronic supplementary material, table S1). For 14 plant species where post-fire reproductive mode was unavailable in the databases, we used the scientific literature (electronic supplementary material, table S1). On conclusion of this research, the post-fire reproductive mode remained unknown for nine plant species (electronic supplementary material, table S1). This process provided baseline information to help make predictions about the potential effects of changing fire regimes on the interactions identified in our review.

As mutualisms vary in their degree of dependency [67], we categorized mutualisms as facultative or obligate. Obligate mutualisms are those where both interactors benefit from the interaction and are solely dependent on each other for survival [67]. Facultative mutualisms are those where both interactors benefit but there is no dependency of either interactor for survival [67]. Given that our review focused on specialized interactions, facultative mutualisms were used to describe interactions where both interactors benefit, but only one interactor is dependent on the other for survival. For facultative mutualisms to be considered specialized, only one interactor need fit the definition of a specialist, while the other interactor may be more generalist in nature (e.g. a pollinator specialized on a single host plant family, while the host plant family is pollinated by other pollinator families). The same conditions applied to commensal and antagonistic interactions where only one interactor was considered specialist. Some species included in our review had undergone binomial nomenclature revisions since publication, and we include the current name while listing the basionym for the original publication in electronic supplementary material, table S1.

Our review revealed 52 cases where fire influenced specialist plant–animal interactions (electronic supplementary material, table S1). These were mutualisms (17 cases), including pollination, dispersal, food and protection provision; commensal interactions (8 cases), including habitat and protection provision; and antagonistic interactions (11 cases), including seed predation, herbivory and parasitism. We also identified specialized multi-faceted interactions that occurred between more than two interactors (16 cases). In our review, articles studied the impacts of either singular fire events (e.g. a wildfire, prescribed fire or experimental burn) or fire regimes (e.g. spatio-temporal patterns of fire) on specialized plant–animal interactions. Articles in our review were published between 1966 and 2021; however, most (*n* = 45) were published since 2004, with only a few (*n* = 4) published prior to 1999. Papers mainly reported data from North America and Central America (*n* = 22) and Australia (*n* = 16), with a small number from Africa (*n* = 5), South America (Brazil and Argentina, *n* = 3), Europe (Spain, *n* = 2) and Asia (South Korea, *n* = 1).

### (a) Plant–animal interactions in fire-prone ecosystems

#### (i) Pollination

We identified nine specialist pollinator interactions, including weevils, moths, bees and wasps, in fire-prone shrublands (electronic supplementary material, table S1). In Mediterranean subtropical shrublands of Spain, the dwarf palm *Chamaerops humilis* (Arecaceae) is primarily pollinated by the weevil *Derelomus chamaeropis* (Curculionidae) [35]. In return for pollination, *C. humilis* provides *D. chamaeropis* with food and habitat in the form of egg deposition and larval development sites in persistent old inflorescences (figure 1) [35,68]. Larval development exerts a fitness cost on the palm by limiting reproductive success; thus, *C. humilis* prevents larval development during fruit development in female inflorescences (figure 1) [69]. Odour mimicry and flowering synchronicity attract weevils to male and female *C. humilis*, despite the lower pollination reward from females (figure 1) [35,70]. Following fire, *C. humilis* resprouts, grows rapidly and resumes flower production the following spring [35,71]. However, *D. chamaeropis* is often less abundant in the immediate post-fire environment owing to fire-related mortality, with recovery reliant on recolonization of burnt sites [35]. In this post-fire stage, *C. humilis* is also pollinated, albeit to a lesser degree, by the sap beetle *Meligethinus pallidulus* (Nitidulidae), protected from fire in stems of *C. humilis* and temporarily replacing the weevil as the primary pollinator [35]. Selection pressure from the weevil affects seed production in *C. humilis* through traits such as flowering synchronicity, which likely feeds back into weevil population dynamics (figure 1). This example shows how co-evolution in a plant–animal interaction is maintained by fire.

Two studies in desert shrublands of North America examined how wildfire influenced interactions between yucca moths in the genera *Tegeticula* and *Parategeticula* (Proxidae), which specialize on pollinating *Yucca* spp. (Asparagaceae) [72,73]. Obligate nursery pollination mutualisms occur between *Yucca brevifolia* and *Tegeticula synthetica* and *Tegeticula antithetica* [74,75], and between *Yucca baccata* and *Tegeticula yuccasella*, with larvae consuming only a fraction of the seeds [74,76]. Agavaceae are known to selectively abscise flowers in response to small pollen loads or self-pollination [76]. Therefore, reinforcing selection for high pollination efficiency but relatively lower egg deposition in yucca moths has been suggested to reduce the risk of flower mortality prior to larval development (figure 1) [76]. High pollination efficiency in yucca moths increases *Yucca* spp. fitness and has led to disinvestment in an ancestral co-pollinator owing to the higher energetic cost to sustain its mutualism [76]. Adult *Y. brevifolia* and *Y. baccata* are killed by fire but have post-fire resprouting capabilities (electronic supplementary material, table S1). However, their post-fire recovery is limited by the low desert precipitation (figure 1) [77,78]. This means that both yucca moth larvae and their host plants are vulnerable to fire-induced mortality while adult moths may survive fire through underground nesting (figure 1) [73]. However, low dispersal ability of adults (e.g. *ca* 8 m dispersal for pollen transfer) [73] coupled with limited post-fire recruitment of *Yucca* spp. could result in local extirpation of yucca moths where frequent fire and drought limit *Yucca* spp. recruitment (figure 1).

In the same North American desert, the shrub *Krameria grayi* (Krameriaceae) provides oil to bees in the genus *Centris* (subgenus *Paracentris*, Apidae) for larval provisioning and nest building (electronic supplementary material, table S1) [73,79,80]. This is a facultative mutualism, with *K. grayi* reliant on *Centris* bees, while the bees are able to collect oil from other plant families [79]. *Krameria grayi* aboveground biomass is killed by fire [73] and he plant resprouts from the root following fire (electronic supplementary material, table S1) [81]. Low post-fire resprouting in *K. grayi* may be attributed to mortality and growth restrictions due to water stress in desert ecosystems or increased root growth at the expense of above ground growth (figure 1) [82,83]. Although adult *Paracentris* bees survive fire by nesting underground [73], limited regeneration of their host species *K. grayi* means that very intense or frequent fires could lead to a local collapse in the bee population where *K. grayi* is the dominant host species (figure 1). Over evolutionary time, oil production in plants has been lost multiple times and, in some cases, was driven by the loss of pollinators [84]. Co-evolution between oil bees and oil flowers has produced specialized morphologies and oil bee behaviours that assist oil collection [85,86]. Thus, selection for oil production in *K. grayi* is likely to be driven by the beneficial effect of the oil on the bee populations [80,85] (figure 1).

In North American temperate sagebrush steppe, *Diadasia enavata* (Apidae) and *Megachile parallela* (Megachilidae) are two oligolectic bee species (i.e. bees with narrow diet specialization) that pollinate wild sunflower, *Helianthus annuus* (Asteraceae) (electronic supplementary material, table S1) [87]. These are facultative mutualisms, with *D. enavata* and *M. parallela* reliant on *H. annuus* while the sunflowers are also pollinated by generalist honeybees *Apis mellifera* and bumblebees *Bombus terrestris* (Apidae) [88,89]. *Diadasia enavata* and *M. parallela* bees nest underground [87], which likely results in their protection during fire (figure 1). However, both bee species were found to be highly sensitive to a large wildfire that razed 121 400 ha of sagebrush steppe in 2010, leaving only small strips of *H. annuus* remaining along roadsides [87]. *Helianthus annuus* does not resprout (electronic supplementary material, table S1) [63], and post-fire recovery is likely reliant on seed dispersal to the burnt area. These bee species are restricted to patches of unburnt habitat during vegetation recovery, leaving them at risk of predation during foraging [87]. Oligolectic bees are also limited in exploiting new hosts as they rely on floral scent recognition and have lower larval fitness when feeding on pollen of other hosts [90,91]. If sunflowers were unable to recover following severe wildfire, local extinction of the bees could follow, as partner switching is unlikely in *Diadasia* bees [92,93]. Sensitivity to large, intense wildfires appears common in specialist pollinator species as their food sources are restricted, resulting in longer post-fire recovery of the interaction [1].

Another hymenopteran pollinator in the same temperate sagebrush steppe is the wasp *Pseudomasaris vespoides* (Vespidae), whose larvae feed on pollen of the forbs *Penstemon* spp. (Plantaginaceae) (electronic supplementary material, table S1) [94]. This is a facultative mutualism, whereby *P. vespoides* relies on *Penstemon* species for food, while *Penstemon* can be pollinated by other insects or hummingbirds [95]. Both *P. vespoides* and *Penstemon cyaneus* are sensitive to fire as the wasps produce nests on rocks and woody stems, exposing them to excessive heat during fire [94]. *Penstemon cyaneus* is killed by high-intensity fire as the basal buds which often confer resprouting capacity are located on the rosette surface, rather than underground (electronic supplementary material, table S1) [94]. Thus, fire intensity would act as a limiting factor for *P. vespoides* nest survival and post-fire resprouting in *P. cyaneus*. If *P. vespoides* pollination was restricted by increasing fire frequency and/or intensity, *Pentstemon* spp. could potentially switch to a hummingbird pollination syndrome [95]. Hummingbirds have traits allowing them to recolonize burnt areas and exploit post-fire flowering [96]. This type of wasp-to-hummingbird pollination transition has occurred numerous times in *Penstemon*, but backwards transitions (hummingbird-to-wasp) have not been recorded [95]. In this ecosystem, more frequent or intense fires could result in local population declines for both species or potential collapse of the interaction, with the wasps being the more at risk owing to their specificity.

Contrary to these fire-sensitive mutualisms, another bee–plant mutualism in the same ecosystem was not harmed by fire [94]. *Micrandrena* (subgenus of *Andrena*, Andrenidae) oligolectic bees pollinate forbs of *Lomatium* spp., including *Lomatium dissectum* (Apiaceae) (electronic supplementary material, table S1) [94]. This is a facultative mutualism, with *Micrandrena* bees reliant on *Lomatium* spp. for pollen, while *L. dissectum* is able to self-pollinate, or be pollinated by other bee species in the Apidae and Halictidae families, albeit with fewer visitors [97,98]. Fire seasonality appears to be important to this interaction because pollination and seed production occur before the fire season [99,100]. However, this interaction appears to involve traits that confer resistance to changes in fire regimes. For example, despite resprouting ability, *L. dissectum* also produces dormant seeds which could sustain the mutualism even under more frequent fires (figure 1) [99]. Furthermore, *Micrandrena* bees nest underground, lowering their susceptibility to mortality during fire (figure 1) [94].

#### (ii) Dispersal and food provision

We identified a single fire-dependent specialist dispersal and food-provisioning interaction from fire-prone shrublands (fynbos) and grasslands of the southwestern Cape, South Africa, where fire-sensitive forests occur among rocky outcrops (electronic supplementary material, table S1) [101]. Fleshy fruit of the rockwood tree, *Heeria argentea* (Anacardiaceae), is dispersed by the generalist frugivore the Namaqua rock rat, *Aethomys namaquensis* (Muridae), which moves *H. argentea* seeds within and between rocky outcrops [101]. This is a specialized facultative mutualism as *H. argentea* is reliant on the rat, not only for dispersal to low fire frequency areas but also for breaking seed dormancy and triggering germination, which otherwise fails without pericarp consumption [101]. The fleshy fruits of *H. argentea* make them sensitive to fire [102], and aboveground biomass of the plants is killed by fire, with no fire-stimulated germination or resprouting (electronic supplementary material, table S1) [101]. The rat consumes only the pericarp of the seed, so seeds are adequately dispersed, while the rat receives a food source [101]. Thus, strong selection on seed pericarp production and seed dormancy in *H. argentea* promotes dispersal by *A. namaquensis* and allows this fire-sensitive forest tree to remain connected across fire-free refugia, within a broader landscape maintained by recurrent fire (figure 1).

#### (iii) Food, habitat and protection provision

We identified eight mutualistic food and protection provision interactions, in the Americas and Africa (electronic supplementary material, table S1) [103–108]. In South American tropical savannah, a mutualistic interaction occurs between the bromeliad *Bromelia balansae* (Bromeliaceae) and the spider *Psecas chapoda* (Salticidae), a predatory carnivore (electronic supplementary material, table S1) [103,109]. Fires are frequent in this system, with plant species typically adapted to fires of a range of intensities (e.g. 500 to 50 000 kW m^−1^ [110]). *Bromelia balansae* can resprout post-fire (electronic supplementary material, table S1) [111] and its rosettes act as a foraging, reproductive and nursery site for the spiders [103,109]. Spider faeces and their prey carcasses are absorbed by bromeliad trichomes, providing nutrients to the plant, and spiders deter herbivores [103,109]. This is a facultative mutualism as *P. chapoda* relies on bromeliaceous species, including *B. balansae*, *Ananas comosus* and *Aechmea distichantha* (Bromeliaceae), for its entire life cycle [103,109,112]. *Bromelia balansae* can gain nutrients in the absence of the spider, but its growth is greater in the presence of the spider [109], likely increasing *B. balansae* survival through recurrent fires. Other Bromeliaceae species, and their associated animals, have shown high mortality following high-intensity wildfires despite the bromeliads' resprouting capacity [113,114]. Therefore, fire intensity is a key environmental parameter influencing the outcome of food provision and shelter interactions (figure 1), with changes in fire intensity disrupting these mutualisms [103].

We identified a number of mutualistic food and protection interactions in Central America, Mexico and Africa between thorn trees (*Vachellia* spp., Fabaceae) and ants of the Formicidae (electronic supplementary material, table S1) [104–107,115,116]. *Vachellia* spp. produce extrafloral nectaries and modified leaflet tips, and swollen thorns which provide food, habitat and protection from fire for ant populations as the thorns do not readily ignite [104,105,115]. These are obligate mutualisms as ants protect the plants from herbivores by attacking them [115]. Ants also protect the plants from fire as their behaviour creates a cleared area around the tree base, reducing fuel load [104–107,115,116]. Ants only consume the leaflet tips and collect nectar from extrafloral nectaries, which limits overgrazing [104]. In the absence of ants, the plants can lose competitive ability against other plant species and suffer severe defoliation from herbivory, with increased mortality, especially post-fire [104,105,107,115,116]. *Vachellia* spp. require intermediate fire frequencies for population turnover and regeneration, with rapid post-fire resprouting and development of thorns, nectaries and leaflet tips, which maintain the mutualistic associations with ant colonies (electronic supplementary material, table S1) [104,107,115]. Production of extrafloral nectaries has been associated with a disinvestment in defence chemical production [115,117]. *Vachellia* spp. are completely reliant on the ants for protection, meaning extrafloral nectary and swollen thorn production are both under strong selection pressure, and high-intensity fire could disrupt this mutualism [104].

### (b) Commensal interactions

#### (i) Habitat or protection provision

We identified eight animal species having a commensal habitat association with spinifex grasses in the genus *Triodia* (Poaceae) in Australia (electronic supplementary material, table S1). Many Australian passerines are habitat specialists relying on spinifex grasslands for nesting and protection from predators (electronic supplementary material, table S1), including (but not limited to) *Amytornis dorotheae* in tropical savannah, *Amytornis woodwardi* in tropical plateau spinifex and *Stipiturus mallee* (Maluridae) in semi-arid mallee shrubland [118–125]. *Triodia* species have both seeding and resprouting post-fire reproductive modes (electronic supplementary material, table S1), but *Triodia* takes a long time to develop high-density tussocks (e.g. 15–30 years post-fire) because of its sclerophyllous leaves and low precipitation in their arid and semi-arid habitat [126–129]. This means that *Triodia*-specialist birds are typically absent from habitat burnt within 15 years [121,122]. Variability in post-fire successional stages, coupled with limited dispersal, causes *A. dorotheae* and *S. mallee* to form distinct metapopulations in otherwise continuous habitat but also puts them at greater risk of mortality from intense fires [118–124]. Local extinctions [124] and failed translocations [130] in *S. mallee* have been driven by fire regimes that shift the ecosystems into a state dominated by early and mid-successional vegetation, a process affecting a range of other species [131].

*Triodia* grasslands cover almost 30% of the Australian continent [61], resulting in many Australian lizards also relying on *Triodia*, including (but not limited to) those in the genera *Ctenotus* (Scincidae), *Ctenophorus* (Agamidae) and *Delma* (Pygopodidae) (electronic supplementary material, table S1) [132–134]. This widespread distribution of *Triodia* has long been thought to be a driver of Australia’s high reptile diversity, as *Triodia* provides the lizards with shelter and with food in the form of termites, which are the main consumers of *Triodia* [135,136]. Some *Triodia*-dependent lizards decline immediately post-fire and take at least 5 years, and often many more, to recover or reach their peak abundance [132–134]. The small marsupial carnivore southern ningaui, *Ningaui yvonneae* (Dasyuridae), is another mid- to late successional *Triodia* specialist from semi-arid mallee shrublands [131,137,138]. *Ningaui yvonneae* uses *Triodia* hummocks and ground litter to forage for invertebrate and vertebrate prey [131,137] and is completely absent from areas burnt within 5 years [132]. Like the grasswrens (*Amytornis*), *Triodia*-specialist reptiles and mammals are sensitive to fire regimes that shift the ecosystem into an early or mid-successional state [131,138].

Given the commensal nature of these relationships, it is unlikely that these *Triodia-*specialist animals pose a strong selection pressure on *Triodia* spp. However, fire return interval is a critical parameter influencing the distribution and abundance of *Triodia* grasses [126] (figure 1), which directly relate to the recovery and persistence of these *Triodia*-specialist animals.

### (c) Antagonistic interactions

#### (i) Seed predation

We identified five specialist antagonistic seed predation interactions in shrublands of Argentina and Spain, and in North American and Australian forests. These specialist seed predators in Argentina and Spain are phytophagous (i.e. plant-feeding) insects that spend their entire life cycles on a single host plant (electronic supplementary material, table S1) [139,140]. Seeds from the climbing plant *Rhynchosia edulis* (Fabaceae) and Mediterranean gorse, *Ulex parviflorus* (Fabaceae), are consumed by the weevils *Acanthoscelides* spp. (Chrysomelidae) and *Exapion fasciolatum* (Apionidae), respectively (electronic supplementary material, table S1) [139,140]. In both cases, phytophagous insect eggs are deposited in flower ovaries and larvae feed on the seed [139,140]. Seeds from the herb *Asphodelus ramosus* (Asphodelaceae) are similarly consumed by the bug *Horistus orientalis* (Miridae), but eggs are deposited inside the inflorescence stalk, with larvae and adults feeding on the leaves, flowers, fruits and seeds (electronic supplementary material, table S1) [140]. In all these cases, fire disrupts the antagonistic interaction by temporarily reducing host plant abundances and seed predators [140]. Plant species respond to fire by triggering post-fire resprouting in *R. edulis* [139], by breaking seed dormancy and stimulating germination in *U. parviflorus* [140], and by triggering flowering in *A. ramosus*, which is otherwise limited in high-density unburnt shrubland (electronic supplementary material, table S1) [140]. Although obligate seeding species, such as *U. parviflorus* (electronic supplementary material, table S1), face immaturity risk under very frequent fire (e.g. every 2 years), they are also limited under fire exclusion, which reduces opportunities for post-fire seed regeneration [140–142]. Thus, short fire return intervals (e.g. every 5 years) would generally favour the plant species of these specialized antagonistic interactions, provided they are longer than the time to maturity (figure 1).

In western North American coniferous forests, the American red squirrel, *Tamiasciurus hudsonicus* (Sciuridae), is a seed predator of the dominant tree species Rocky Mountain lodgepole pine, *Pinus contorta latifolia* (Pinaceae) [55,56]. Fires in this ecosystem occur every 75−300 years and fire prevents successional progression to spruce–fir climax communities which have lower fire frequencies [143,144]. *Pinus contorta latifolia* is an obligate seeder, which releases seed from serotinous cones following fire, resulting in mass recruitment events that produce dense, even-aged stands [63,144–146]. However, *P. contorta latifolia* requires *ca* 70 years to develop a substantial seed bank, placing this tree species under immaturity risk if fires occur more frequently than its time to maturity [147,148]. Increasing fire frequencies also place selection pressure on serotiny, a heritable trait where cones remain closed until an environmental trigger causes them to open, as it confers seed survival during fire (figure 1) [55,56]. However, this trait is also under strong negative selection pressure from pre-dispersal seed predation by *T. hudsonicus* owing to its selective harvesting of serotinous cones (figure 1) [55,56]. Any change in fire frequency can cause shifts in the vegetation community, with projections showing transitions from forests to shrublands under increasing fire frequency [143,149], lowering food resources and the dense canopies characteristic of *T. hudsonicus* habitat [150]. Conversely, a decrease in fire frequency allowing the spruce–fire climax community [143,149] would also decrease food resource availability for *T. hudsonicus* owing to low *P. contorta latifolia* population turnover as stands senesce [148]. This interaction results in spatial variation in serotiny, demonstrating how fire influences plant and animal traits under selection, which can subsequently feed back into population processes.

Another specialist seed predator is the glossy black cockatoo, *Calyptorhynchus lathami* (Cacatuidae) [151,152], which feeds solely on the seeds from 12 serotinous tree species in the Casuarinaceae family (she-oaks) in woodlands and forests of eastern Australia [153–155]. While two glossy black cockatoo subspecies exist, *C. lathami halmaturinus* is, to our knowledge, the only subspecies for which the plant–animal interation has been studied in relation to fire. On Kangaroo Island, South Australia, *C. lathami halmaturinus* feeds exclusively on the she-oak *Allocasuarina verticillata* [154]. Very little is known about the effect of *C. lathami* predation on she-oaks themselves, but seed predation by cockatoos exerts strong selection pressure on other Australian serotinous species [156,157]. *Allocasuarina verticillata* is a facultative resprouter (electronic supplementary material, table S1) [154,158], but has poor resprouting and seed regeneration in the absence of fire [154]. In *A. verticillata*, fruits appear at 5−10 years of age, but seedbanks take 20 years to accumulate adequately for recruitment [154], meaning frequent fire (e.g. every 5−10 years) limits she-oak recovery. The cockatoos display variable geographical selection of seed, owing to tight energy budgets, avoiding recently burnt (e.g. <10 years post-fire) and long-unburnt stands (e.g. >60 years post-fire) as a result of low cone abundance or low cone quality, respectively [154,155]. Thus, fire frequency is a critical factor in maintaining food sources for the glossy black cockatoo (figure 1). *Calyptorhynchus lathami halmaturinus* is currently limited to Kangaroo Island, where increases in large high-intensity and frequent wildfires have limited food availability in an already limited foraging habitat [154]. High-intensity fires have caused regional population declines in *C. lathami halmaturinus*, highlighting the importance of maintaining unburned regions for persistence in this specialized seed predator [154]. At the other extreme, if fire is excluded from an ecosystem for more than 60 years, reductions in recruitment and canopy seedbank abundance could lower food resource availability for *C. lathami* [154,155].

Fire controls plant abundances and, thus, food resources essential to these specialist antagonistic seed predators (figure 1). Single fire events can temporarily reduce host plant abundances and, thus, seed predation, allowing plant recruitment processes under lower biotic pressures. Partner switching, whereby an animal species switches its primary food source plant, has been documented in response to food resource fluctuations in non-fire research [159,160]. Whether a species such as *C. lathami* could switch permanently to alternative food sources is unknown, but this species has been observed to feed on other species in response to temporary food shortages [155,161]. If partner switching did not occur, an abrupt fire regime change is more likely to cause subsequent collapses in such antagonistic plant–animal interactions.

#### (ii) Herbivory

We identified six specialist antagonistic herbivore interactions involving larval butterflies and adult beetles and bugs (electronic supplementary material, table S1). Adult butterflies of specialist larval butterflies identified in these cases are generalist pollinators [162–164]. Therefore, these interactions were considered antagonistic only at the larval stage, as larval herbivory results in stem defoliation, which limits plant growth [165,166]. In North American temperate shrublands, grasslands and forests, perennial forbs in the *Viola* genus (Violaceae), including *Viola pedata, Viola pedatifida*, *Viola sororia*, *Viola sagittata* and *Viola bicolor*, are larval host plants for *Speyeria idalia* and *Speyeria cybele* (Nymphalidae) (electronic supplementary material, table S1) [165,167–175]. *Viola sororia* is the only known resprouter of these *Viola* species (electronic supplementary material, table S1), but other *Viola* species are known to resprout [64], and some increase in abundance with increasing fire frequency [169]. Butterfly larvae of *Cercyonis pegala* (Nymphalidae) specialize on *Tridens flavus* (Poaceae), a post-fire resprouter, in North American temperate prairie (electronic supplementary material, table S1) [167,170,171]. *Polygonia c-aureum* (Nymphalidae) is another larval specialist butterfly, found in subtropical forests and grasslands of South Korea [176]. Larvae of this species feed on the vines *Humulus japonicus* and *Humulus lupulus* (Cannabaceae), which have increased growth post-fire owing to gap creation [176] (electronic supplementary material, table S1). Resprouting is important for these host plants, conferring plant re-establishment, and for the persistence of butterfly populations, by increasing nectar source abundance and egg deposition substrate for adults (figure 1) [167,174,177]. More frequent fire might lead to higher rates of herbivory by reducing habitat complexity, promoting butterfly larvae ability to locate host plants [178].

We identified one coleopteran and one hemipteran species as specialist herbivores in fire-maintained North American ecosystems. In temperate sagebrush steppe, the beetle *Trirhabda lewisii* (Chrysomelidae) feeds on the foliage of the shrub *Ericameria nauseosa* (Asteraceae) and can completely defoliate the shrubs, sometimes causing plant death (electronic supplementary material, table S1) [179]. In temperate oak savannah, the oak lace bug, *Corythucha arcuata* (Tingidae), is entirely reliant on the oak tree *Quercus macrocarpa* (Fagaceae) for its life cycle, feeding on leaf mesophyll (electronic supplementary material, table S1) [180,181]. Feeding by *C. arcuata* results in leaf discoloration [180], reducing the oak’s photosynthetic capacity [182]. In both cases, the plant species can resprout post-fire (electronic supplementary material, table S1), with fires temporarily disrupting these antagonistic herbivorous interactions owing to insect mortality [179,180,183,184]. However, fire can produce more appealing plant material for *T. lewisii*, resulting in increased herbivory and, therefore, post-fire mortality in *E. nauseosa* [179]. *Quercus macrocarpa* usually survives low-intensity fires by resprouting, with canopy gaps produced by fire increasing its growth, but also leaf quality for *C. arcuata* [180,184]. Consequently, fires can result in higher densities of both of these specialist herbivores owing to higher-quality plant material [179,180]. Frequent but low-intensity patchy fires, which do not compromise the plant’s capacity to reach maturity (figure 1), can maintain high-quality food resources for specialist herbivores by triggering regeneration of their food plants. Outside of this North American system, *C. arcuata* can feed on other plant families [181], suggesting an ability to shift host plants if fire regimes become unfavourable (e.g. increases in fire frequency). If plant hosts were released from their enemies, resource allocation could shift from plant defence to other population processes such as individual growth [185].

### (d) Multi-faceted interactions

#### (i) Food and protection interactions

We identified two multi-faceted food and protection interactions between butterfly species, their larval host plant, and ant species (electronic supplementary material, table S1). In North American temperate upland prairie, *Icaricia icarioides fenderi* (Lycaenidae) larvae feed on the leguminous lupines *Lupinus sulphureus*, *L. sulphureus kincaidii*, *Lupinus argenteus laxiflorus* and *Lupinus arbustus* (Fabaceae), which also act as a nectar source for adult butterflies (electronic supplementary material, table S1) [186–188]. The association with *Lupinus* spp*.* was considered specialized only at the larval stage as adult butterflies collect nectar from other plant families [189] and these *Lupinus* spp*.* are also pollinated by bees and flies [190]. Associations between *Lupinus* spp*.* and *I. icarioides fenderi* larvae are antagonistic as the larvae feed on young leaves and apical meristems [187,191], reducing plant growth. *Icaricia icarioides fenderi* also has a facultative mutualistic association with ants (Formicidae) as their myrmecophilous organs provide nutrients to the ants, while the ants protect the butterfly larvae from predation and parasitism [186]. In this case, the effect of fire on the ants was not directly investigated, but the ants likely seek refuge from fire in underground nests, conferring survival [105]. While the ant–butterfly larva association in these species is only facultative, this mutualism allows butterfly larvae to inhabit enemy-free space [192]. The butterfly larvae are killed by fire [186,188], but adult butterflies quickly recolonize burnt areas and increase their reproduction in the year after fire [187], suggesting resilience of *I. icarioides fenderi* to certain fire regimes. *Lupinus sulphureus* is a resprouter, and both post-fire resprouting and seeding have been recorded in *Lupinus* spp. (electronic supplementary material, table S1) [193,194]. Thus, the *Lupinus* spp. on which *I. icarioides fenderi* rely are likely to resprout, allowing fast re-establishment of these interactions. Favourable fire regimes (e.g. burning a proportion of the habitat each year) are essential for maintaining a mosaic of regenerating burnt and unburnt habitat for the persistence of this specialized interaction (figure 1) [187,188].

In Mexican subtropical forests the shrub *Croton repens* (Euphorbiaceae), butterfly *Anatole rossi* (Riodinidae) and ant *Camponotus atriceps* (Formicidae) form specialized interactions (electronic supplementary material, table S1) [195]. Adult butterflies almost exclusively rely on *C. repens* for nectar, but have been observed feeding on *Ruellia* spp. (Acanthaceae) and *Calea longipedicellata* (Asteraceae) [195]. It is unknown if other species may act as pollinators for *C. repens*, but other *Croton* species have generalist wasp and bee pollinators [196]. *Anatole rossi* larvae completely defoliate *C. repens* and feed on new buds, limiting growth [195], indicating an antagonistic interaction. However, these shrubs can produce new growth quickly in the absence of *A. rossi* larvae [195]. *Anatole rossi* larvae possess myrmecophilous organs, which promote ant tending through a facultative mutualism, as ants also collect honeydew from other plants, but tending by *C. atriceps* is essential for *A. rossi* larvae survival [195]. Production of nectar and myrmecophilous organs is energetically costly for *A. rossi* larvae [192], and the association with ants for larval survival likely drives selection on these traits (figure 1). *Anatole rossi* larvae are protected from predation and fire as ants coerce them into underground tunnels along tap roots of *C. repens* [195]. Fire is required for *C. repens*, and the maintenance of the interaction as fire triggers resprouting reduces competition and provides egg deposition sites for female butterflies [195]. However, if fires becomes more intense and severe, the underground tunnels might be too shallow to protect *A. rossi* larvae and ants, resulting in mortality (figure 1) [195].

### (ii) Antagonistic parasitism—mutualistic dispersal and food interactions

We identified 14 multi-faceted antagonistic parasitism–mutualistic dispersal and food interactions, namely interactions occurring between host-specific mistletoes (Loranthaceae), their host plant, and avian vectors occurring in Brazil and Australia (electronic supplementary material, table S1) [197–199]. Mistletoes are host-specific hemi-parasites (Santalales) that attach to hosts via a modified root (i.e. haustorium) and can negatively impact their host by limiting water and nutrient uptake [200]. Mistletoes are reliant on numerous bird species for dispersal [197,198,201], but in Australia the mistletoe bird, *Dicaeum hirundinaceum* (Dicaeidae), is the most effective disperser. The impacts of fire on mistletoe-dispersing birds will, therefore, have flow-on effects for the parasite–host interaction [198]. Many mistletoe species occur in fire-prone environments, and most of their hosts plants have the capacity to resprout post-fire, and some also have capacity for post-fire seeding (electronic supplementary material, table S1) [63,64,198]. This facilitates quick re-establishment of host plants following fire and likely also mistletoe parasitism. However, three mistletoe host plants do not resprout (*Acacia monticola*, *Acacia xiphophylla* and *Vatairea macrocarpa* (Fabaceae)) and few mistletoe host plants are capable of post-fire seeding (e.g. *Acacia aneura*, *Acacia monticola*, *Acacia xiphophylla*, *Lysiphyllum cunninghamii* (Fabaceae) and *Grevillea wickhamii* (Proteaceae)), leaving these mistletoes more susceptible to increasing fire frequencies and intensities (electronic supplementary material, table S1) [202]. Mistletoes generally lack the ability to resprout and would need to recolonize burnt areas through seed dispersal (electronic supplementary material, table S1) [197–199]. One mistletoe species in Australia, *Amyema sanguinea sanguinea* (Loranthaceae), is able to resprout from the haustorium but only when of a sufficient size to have thick bark providing protection from fire [198,199]. Thus, mistletoe size is an important characteristic related to the persistence of the parasitic interaction through fire [197]. Fire seasonality is also important, as fires prior to flowering or seed dispersal can inhibit reproduction in mistletoes, while fire prior to the wet season allows hosts, and their parasites, to recover quickly [197]. In some cases, fire can increase mistletoe recruitment by reducing vegetation density and increasing light availability for germination and development [197–199]. However, fires that are too frequent (e.g. every 2 years) or intense are likely to cause local population declines as mistletoe could be killed before seed dispersal by mistletoe-dispersing birds [197–199].

The mistletoe bird, *D. hirundinaceum*, has an alimentary canal adapted specifically for a diet based on mistletoe fruits, and defaecation behaviour that ensures mistletoe seeds adhere to host plant branches [198]. The interaction between *D. hirundinaceum* and mistletoe is a specialized facultative mutualism in Australia as mistletoe berries compose the majority of their diet [203], but mistletoes are dispersed by other bird species [197,198,201]. Immediate post-fire dispersal of mistletoe seeds in Australia might also rely on more generalist avian vectors (e.g. *Acanthagenys rufogularis*, *Canopophila whitei*, *Meliphaga lewinii* (Meliphagidae) [198], and *Zosterops lateralis* (Zosteropidae) [201]), as a lack of mistletoe food sources results in post-fire declines of *D. hirundinaceum* [204]. However, mistletoe recruitment may decline if fires are frequent (e.g. every 2 years), as other avian vectors may not disperse mistletoe at high rates and lack the specialized physiology and defaecation behaviour of *D. hirundinaceum* [204]. Thus, complex interactions occur between mistletoes and fire, with fire regimes of low intensity and frequency (e.g. return interval >2 years) likely to promote mistletoe abundance, confer survival on their host plants and allow dispersal by avian vectors including the specialist *D. hirundinaceum* (figure 1).

## 3. Eco-evolutionary dynamics in fire-dependent interactions

Eco-evolutionary feedbacks occur when ecological processes (e.g. demographic change and species interactions) influence evolutionary changes (e.g. trait and allele frequencies), subsequently feeding back into the ecological process (and *vice versa*) [205,206]. A number of criteria must be met for eco-evolutionary dynamics to be demonstrated. First, natural selection or evolution must be shown to occur on ecological time scales (tens of generations or fewer) [207,208]. Second, the evolutionary change must feed back into the ecological change via its influence on the environment, or the population dynamics of the plant or animal involved in the interaction [207,209]. Stabilizing (negative) eco-evolutionary feedbacks occur when directional changes in ecological or evolutionary processes trigger a response from a species or environment that forces a negative response and subsequently maintains the ecosystem at a stable state [210,211]. Fire regimes that occur within the range of variability under which the plant–animal interaction evolved can result in stabilizing eco-evolutionary feedbacks. In many ecosystems, fire controls plant population turnover and promotes plant reproduction, which stabilizes specialized plant–animal interactions such as pollination [1,212]. Fire can also limit antagonistic plant–animal interactions, stabilizing populations by reducing seed predation [140]. Reinforcing (positive) feedbacks occur when a species forces a response in the environment that intensifies a change in population dynamics, leading to local adaptation and further directional environmental change [210,211]. A famous example of a reinforcing feedback implicated in fire regime shifts is the invasion of high-biomass grasses [10,11,213]. When invasive grasses increase biomass and flammability, fire activity can promote further invasion [10,11,213]. Traits promoting fire tolerance and flammability in such grasses have evolved beyond the variability observed in their native range [214,215], suggesting that rapid evolutionary changes can feed back into fire regimes.

Both stabilizing and reinforcing eco-evolutionary feedbacks might be important in plant–animal interactions, if the interaction modifies the environment, and the environment influences the interaction through selection on species traits [51,53,216]. For mutualisms, eco-evolutionary feedbacks generally result in co-evolution. However, partner switching, interaction type switching (e.g. mutualism breakdown, resulting in a switch to antagonism) and co-extinction have been documented when the evolutionary rate of one interaction partner is slow, resulting in reduced fitness of the other interaction partner [51,53]. Eco-evolutionary feedbacks in commensalisms have also resulted in interaction type switches (e.g. to mutualism or antagonism) [52]. However, these can be evolutionary endpoints for the commensal interaction partner if it does not respond in a directional manner that matches their interactor's evolution [52]. Eco-evolutionary feedbacks in antagonistic interactions can result in a range of outcomes, including antagonism release, for example where predators fail to evolve and prey are released from predation [217,218]. Co-evolution of antagonisms can also strengthen the interaction [217,218]. As in other interaction types, co-extinction of specialist predators can occur when the prey has low evolutionary potential, leading to resource limitations for the predator [217,218].

Research has revealed how fire-induced plant population dynamics can feed back into the fire regime by changing ecosystem structure and strengthening selection on plants and animals in fire-prone ecosystems (e.g. [25,219,220]). Animals are often implicated in these dynamics, especially in the case of grazing herbivores [221,222]. Grazing pressures and fire frequency effects on plants are coupled, with preferential grazing in immediate post-fire environments resulting in biomass reductions, further supressing fire [221,222]. When grazing pressure is low, biomass increases and promotes fire occurrence and intensity [221,222], which can also determine where and how herbivores graze [221]. Few studies, however, have explicitly investigated how fire influences eco-evolutionary dynamics in plant–animal interactions, beyond vegetation structure. Exemplary research on this topic is demonstrated by the study of serotiny in lodgepole pine, *P. contorta latifolia*, and the seed predator American red squirrel, *T. hudsonicus* [55,56]. Conflicting directional selection occurs in this plant–animal interaction: fire drives selection for serotiny, and pre-dispersal seed predation by the squirrel drives selection against serotiny [55,56]. Selection against serotiny in *P. contorta latifolia* from seed predation is stronger than selection for serotiny from fire, but only where *T. hudsonicus* is abundant [55,56], resulting in landscape-level variation in serotiny expression. This state-of-the-art research can help us identify the potential for such dynamics in other plant–animal interactions from fire-prone ecosystems. However, few studies have investigated fire-related eco-evolutionary dynamics in as much detail as the squirrel–pine example.

## 4. Conclusion

Research on specialist plant–animal interactions in fire-prone ecosystems is a relatively new field, but given current biodiversity declines as a result of global change it is vital to understand these interactions. We identified a number of specialized mutualistic and antagonistic interactions that affect, and are affected by, variation in fire regimes. Commensal interactions were the most under-represented interaction type in our review and all of them represented animal species specializing on a single plant genus. This was probably because less is known about commensalism in general [52] and also because it was difficult to identify specialist relationships from fire-prone ecosystems. Regardless, drawing together literature on specialized plant–animal interactions allowed us to identify how fire regime changes impact these interactions. This differs from previous work on plant and animal traits generally in fire-prone ecosystems (e.g. [22,23,27]), as we identified key plant and animal traits that critically underlie specialist plant–animal interactions (figure 1). For plants these traits include: reproductive mode, time to maturity, morphology and phenology; and for animals: dispersal ability, nesting substrate, egg deposition substrate, specialized physiology, plant consumption behaviours and pollination behaviours. As managing fire for conservation outcomes is critical, this information could be used to adapt management plans to maximize their suitability for the greatest biodiversity.

While we found widespread evidence for traits involved in plant–animal interactions under strong selection pressure (e.g. post-fire reproduction mode), very few studies have demonstrated fire-driven evolution on ecological time scales in plant–animal interactions. In our review, the reinforcing nature of fire on plant–animal interactions was usually identified to be rapid and result in abrupt changes in the state of the ecosystem. There is evidence that the impact of evolution increases with increasing interaction strength for antagonisms and with decreasing interaction strength for mutualisms [223]. Our review identified more traits involved in mutualisms as being subject to strong selection pressures (figure 1) than those for antagonisms. However, this is more likely a result of the limited number of studies published on this research topic than a biological outcome. Thus, it is clear that more research is required to understand evolutionary changes on short time scales. Key to understanding plant–animal interactions in fire-prone ecosystems and the potential eco-evolutionary feedbacks of fire on these interactions is a detailed knowledge of plant and animal traits involved in fire responses. Our compilation of these traits (figure 1) represents what has been researched to date, rather than a comprehensive list of the traits involved. In some of these specialized interactions, we noted a bias toward focusing on the impacts that fire has on the animal species, rather than the interaction itself. Given that plants are the foundation of animal habitat, understanding plant responses is necessary for any study on plant–animal interactions. However, future research in this area would benefit from reporting the interactive effects of the animal species and fire on the plant species post-fire recovery as this would aid effective conservation and management practices.

## Data Availability

This article has no additional data.
